# LCS-TA to identify similar fragments in RNA 3D structures

**DOI:** 10.1186/s12859-017-1867-6

**Published:** 2017-10-23

**Authors:** Jakub Wiedemann, Tomasz Zok, Maciej Milostan, Marta Szachniuk

**Affiliations:** 1 Institute of Computing Science & European Centre for Bioinformatics and Genomics, Poznan University of Technology, Piotrowo 2, 60-965 Poznan, Poland; 2 Poznan Supercomputing and Networking Center, Jana Pawla II 10, 61-139 Poznan, Poland; 3 Institute of Bioorganic Chemistry, Polish Academy of Sciences, Noskowskiego 12/14, 61-704 Poznan, Poland

**Keywords:** RNA 3D structure, Structure comparison, Local similarity, Torsion angles

## Abstract

**Background:**

In modern structural bioinformatics, comparison of molecular structures aimed to identify and assess similarities and differences between them is one of the most commonly performed procedures. It gives the basis for evaluation of in silico predicted models. It constitutes the preliminary step in searching for structural motifs. In particular, it supports tracing the molecular evolution. Faced with an ever-increasing amount of available structural data, researchers need a range of methods enabling comparative analysis of the structures from either global or local perspective.

**Results:**

Herein, we present a new, superposition-independent method which processes pairs of RNA 3D structures to identify their local similarities. The similarity is considered in the context of structure bending and bonds’ rotation which are described by torsion angles. In the analyzed RNA structures, the method finds the longest continuous segments that show similar torsion within a user-defined threshold. The length of the segment is provided as local similarity measure. The method has been implemented as LCS-TA algorithm (Longest Continuous Segments in Torsion Angle space) and is incorporated into our MCQ4Structures application, freely available for download from http://www.cs.put.poznan.pl/tzok/mcq/.

**Conclusions:**

The presented approach ties torsion-angle-based method of structure analysis with the idea of local similarity identification by handling continuous 3D structure segments. The first method, implemented in MCQ4Structures, has been successfully utilized in RNA-Puzzles initiative. The second one, originally applied in Euclidean space, is a component of LGA (Local-Global Alignment) algorithm commonly used in assessing protein models submitted to CASP. This unique combination of concepts implemented in LCS-TA provides a new perspective on structure quality assessment in local and quantitative aspect. A series of computational experiments show the first results of applying our method to comparison of RNA 3D models. LCS-TA can be used for identifying strengths and weaknesses in the prediction of RNA tertiary structures.

**Electronic supplementary material:**

The online version of this article (10.1186/s12859-017-1867-6) contains supplementary material, which is available to authorized users.

## Background

A comparison of contents stored in NCBI Reference Sequence Database (RefSeq) [1] and Protein Data Bank (PDB) [2] brings to a conclusion that there is a large, ever-widening gap between the numbers of known sequences and structures of biomolecules. Today, this gap is being filled with the use of computational methods that address the problem of RNA and protein 3D structure prediction. Following that, a necessity to estimate the quality of computational models and fidelity of predictors arises. Since the 1990s, CASP (Critical Assessment of protein Structure Prediction) experiment has taken the challenge of assessing protein structure prediction [3]. RNA-Puzzles initiative launched in 2011 and drawing on the solutions implemented in CASP, followed to support the RNA community [4, 5]. Both experiments have significantly contributed to a development of measures and methods for validation and assessment of 3D structure models predicted in silico [6]. The resulting algorithms have been applied not only in the evaluation of predicted proteins and RNAs. They are also used for validation and analysis of experimentally solved structures, clustering 3D models, identification of structure motifs, tracking conformational changes, exploring the sequence-structure relationship, etc. [6–14].

RNA-Puzzles, a collective experiment for blind RNA structure prediction, uses the following approaches to assess submitted RNA 3D models: (i) Root Mean Square Deviation (RMSD), (ii) Interaction Network Fidelity (INF) [15], (iii) Deformation Index (DI), (iv) Clash score by MolProbity [16], and (v) Mean of Circular Quantities (MCQ) [17]. Except that, a few other RNA evaluation methods have been developed and applied in various projects [8, 18]. All of them relate to various attributes of the considered RNA 3D structures, but their common feature is that the structures are mainly evaluated globally. Similarly, most structure assessment methods in CASP treat protein models globally, and only a few touch an aspect of local similarity. Such approach is fully understood and seems sufficient when we deal with the evaluation and ranking of many models submitted to the competition. However, when analyzing individual structures, finding their strengths and weaknesses, comparing substructures, or identifying motifs, a local assessment is necessary. In such cases, local evaluation of the 3D model complements global analysis and significantly enhances our knowledge of the structure.

So far, one approach has been proposed to enable a local view on predicted RNA 3D model compared to the target structure. It is based on a concept of spheres built along RNA backbone and providing the scene for preview and RMSD-based evaluation of sphere-enclosed atom subsets. It has been first implemented as a standalone application named RNAlyzer [8], and later released as RNAssess webserver [19]. In the case of proteins, Local-Global Alignment (LGA) is one of the most common approaches enabling local analysis [20]. LGA comprises two methods, Longest Continuous Segments (LCS) and Global Distance Test (GDT). The first one identifies the longest continual fragment within predicted protein structure which – compared to the target – has the RMSD below a given threshold. The second method computes the percentage of residues fitting below predefined distance cut-off. LGA is the reference method used to evaluate protein structures in CASP.

The methods mentioned in the previous paragraph operate in Euclidean space where each structure is represented as a set of atoms with coordinates in the Cartesian system. As all other approaches which consider molecule structures in Euclidean space and apply RMSD-based evaluation, they deal with the computationally demanding problem of optimum 3D structure alignment. This problem can be omitted when switching to the space of torsion angles. The 3D structure of RNA can be represented by a set of eight torsion angles that describe the course of its backbone and arrangement of the bases. Such representation makes a comparison of structures independent of their alignment in space and simplifies the computation. This concept has been followed in MCQ4Structures method [17] that expresses structure similarity as Mean of Circular Quantities (MCQ).

Here, we propose a new method that integrates a concept of RNA 3D structure comparison in the space of torsion angles [17] with the idea of identifying longest continuous segments displaying local similarity [20]. Two segments are considered similar if their MCQ value is below the predefined threshold. The method has been implemented as LCS-TA algorithm (Longest Continuous Segments in Torsion Angle space) and incorporated into MCQ4Structures software. It is freely available at http://www.cs.put.poznan.pl/tzok/mcq/.

## Methods

LCS-TA has been designed as the local similarity measure. It aims to compare two RNA 3D structures, *S* (structure of the target) and *S′* (structure of the model), and identify similar fragments within them. It runs either in sequence-independent or sequence-dependent mode. In the first mode, the compared structures can have different lengths, and the relationship between their residues can be unknown. Thus, no preliminary analysis of the sequences of *S* and *S′* is required here. In the second mode, the method processes structures of the same length. LCS-TA operates in the space of torsion angles, so it is superposition-independent and does not involve finding the optimum alignment of structures. The method scans both structures stepwise along their backbones and uses a moving search window to select segments for a comparison. In this routine, a divide and conquer formula is followed to determine the window size in each step. For a pair of window-highlighted segments, LCS-TA computes MCQ value over a set of torsion angles related to the segments. Next, it checks whether the MCQ value is below the threshold. At the output, LCS-TA provides the length of the longest continuous segment satisfying similarity condition (i.e., fitting below the threshold) and segment location (its first and last residue numbers). The resulting segment’s length (referred to as LCS) is the measure of local similarity. Both components of the method, that is divide and conquer procedure and MCQ-based measure, are described in the following paragraphs.

### Divide and conquer procedure

**Fig. 1:**
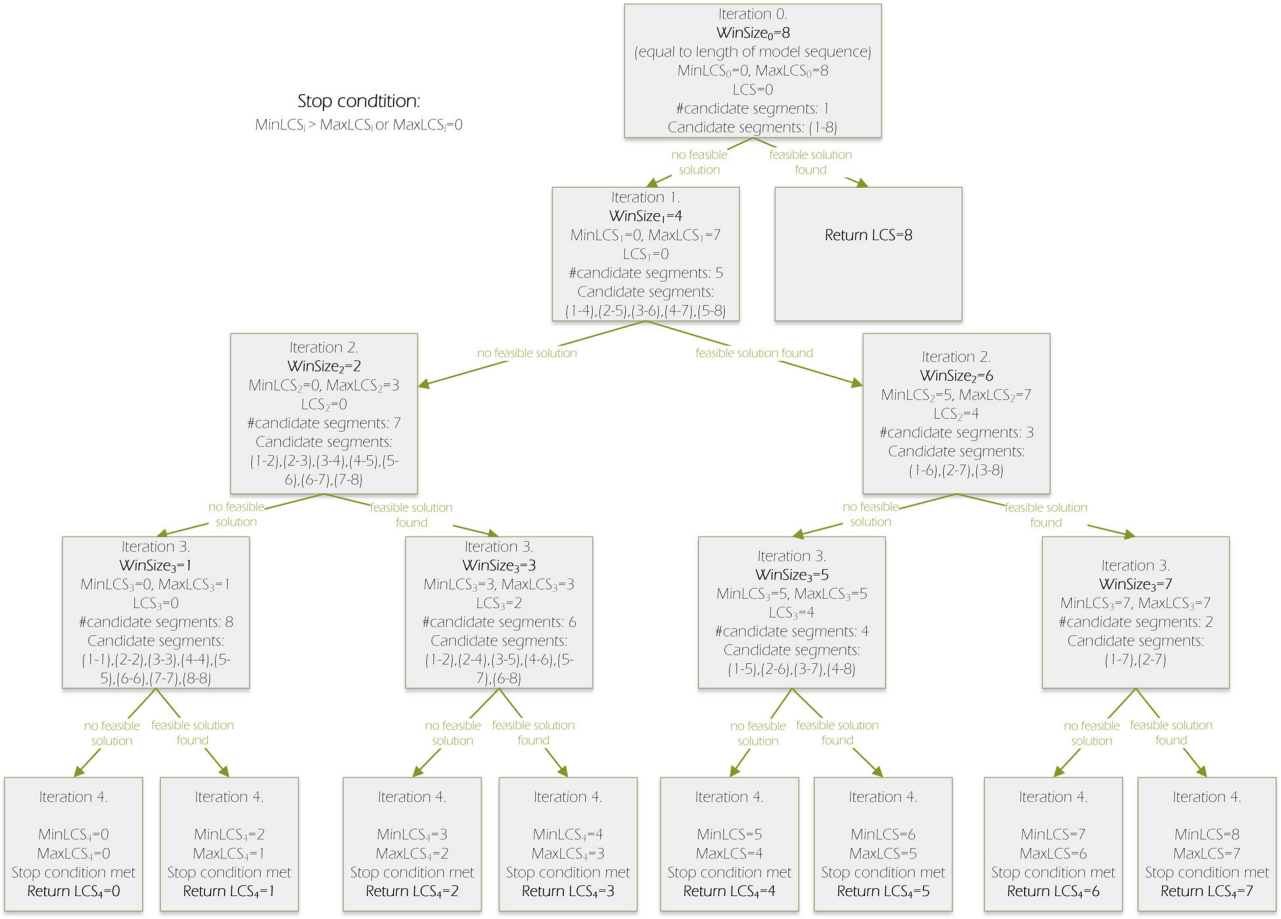
Example recursion tree in LCS-TA algorithm

Divide and conquer (D&C) is a technique used to optimize the process of solving the problem by recursively splitting it into smaller subproblems and using their solutions to build the solution of the input problem. In our method, we apply D&C approach to determine lengths of the search window in consecutive steps of the algorithm. The example recursion tree visualizing divide-and-conquer-driven computation in LCS-TA algorithm is presented in Fig. 1.

The initial window size in LCS-TA is equal to the number *n* of residues in the predicted model (*WinSize* = *n*). In each iteration, the algorithm checks whether a feasible solution (namely continuous segment with MCQ below the threshold) exists for current window size. In the case of a negative result, *WinSize* is divided by 2 (and rounded up to the least succeeding integer). Otherwise, it is incremented to a value halfway between current size and *WinSize* of grandparent iteration (i.e., iteration *i*-2, where *i* is the order number of current iteration) except the first iteration where *n-1* is taken as an upper bound of *WinSize*. Next, the computation runs recursively for both sizes of the search window, thus branching into two subproblems. The algorithm stops if further reduction of the window size is impossible (*WinSize* = 1) and all possible solutions for that *WinSize* value have been checked, or if the optimum solution is found. Such computation pattern, known as binary tree recursion, is one of the most commonly used in the implementation of the D&C method. Its time complexity is O(log_2_ *n*), where *n* is the instance size (in our problem *n* is the number of residues in *S′* – structure of predicted model).

### MCQ-based measure

The MCQ-based distance measure has been developed for trigonometric representation of the molecule 3D structure [17]. In this representation, a shape of every RNA residue is described by eight torsion angles from the set *T* = {α, β, γ, δ, ε, ζ, *P*, χ}. Each torsion angle in RNA molecule is defined by atom quadruple (the details can be found in [17, 21]) and determines rotation around particular chemical bond. It is computed as a dihedral angle between two planes defined by a pair of overlapping atom triples. Having a chain A-B-C-D of four atoms, we can easily determine the torsion angle between the plane passing through A, B, C, and the plane passing through B, C, D.

When the RNA structure is composed of *n* residues, then its trigonometric representation is a matrix containing 8*n* values of torsion angles *t*_*ij*_, where *i* = 1,...,*n*, *j* = 1,...,|*T*|, and *T* is a set of torsion angles defined for RNA (*t*_*ij*_ is torsion angle of type *j* within residue *i*). To measure the distance between two structures, *S* and *S′*, of equal length (*n* residues), given in trigonometric representations, we apply formula (1) for computing mean of circular quantities [17]:

1 $$ \mathrm{MCQ}\left(S,{S}^{\prime}\right)=\arctan \left({\sum}_{i=1}^n{\sum}_{j=1}^{\left|T\right|}\sin \varDelta \left({t}_{ij},{t}_{ij}^{\prime}\right),{\sum}_{i=1}^n{\sum}_{j=1}^{\left|T\right|}\cos \varDelta \left({t}_{ij},{t}_{ij}^{\prime}\right)\right) $$

The two-argument arctan(*y*, *x*) is used to distinguish results from the whole range [−π; π). This is possible, because the function calculates angle value from the positive X half-axis to the vector between points (0, 0) and (*x*, *y*) in a Cartesian coordinate system. In particular, this means that, unlike one-argument $$ \arctan \left(\raisebox{1ex}{$y$}\!\left/ \!\raisebox{-1ex}{$x$}\right.\right) $$ the two-argument variant is well-defined for x = 0 and in general arctan(*y*, *x*) ≠ arctan(−*y*, −*x*) which is not true for one-argument function.

In formula (1), the following function is used to obtain the distance between two angles:

2 $$ \varDelta \left(t,{t}^{\prime}\right)=\left\{\begin{array}{ll}0\hfill & \mathrm{If}\ t\ \mathrm{and}\ {t}^{\prime }\ \mathrm{are}\ \mathrm{undefined}\hfill \\ {}\uppi \hfill & \mathrm{if}\  \mathrm{either}\ t\ \mathrm{or}\ {t}^{\prime }\ \mathrm{is}\  \mathrm{undefined}\hfill \\ {}\min \left\{\mathrm{diff}\left(t,{t}^{\prime}\right),2\uppi \hbox{-} \mathrm{diff}\left(t,{t}^{\prime}\right)\right\}\hfill & \mathrm{otherwise}\hfill \end{array}\right. $$

Where

3 $$ \mathrm{diff}\left(t,{t}^{\prime}\right)=\left|\operatorname{mod}(t)\hbox{-} \operatorname{mod}\left({t}^{\prime}\right)\right| $$

and

4 $$ \operatorname{mod}(t)=\left(t+2\pi \right)\ \mathrm{modulo}\ 2\uppi $$

MCQ has been defined as a distance measure, and it shows the dissimilarity of two three-dimensional structures of the same length. Thus, the greater is its value, the more the two structures differ. And accordingly, the smaller the MCQ value, the greater is the similarity of compared structures.

It should be noted, that set *T* of torsion angles defined for RNA originally contained eight types of angles. However, MCQ is flexible, and any subset of *T* can be used to measure it. For example, if the user is interested to consider ribose ring only, then MCQ can be computed involving pseudotorsion angle *P* (or, alternatively, τ_0_, τ_1_, τ_2_, τ_3_, τ_4_ angles). In the presented version of the algorithm we use original set *T* = {α, β, γ, δ, ε, ζ, *P*, χ}.

Finally, let us add that originally MCQ value is computed in radians. In our application, it is next converted into degrees and so presented to the user.

### LCS-TA algorithm

The LCS-TA algorithm compares two RNA 3D structures (hereby referred to as the target and the model) provided in PDB or mmCIF file formats. At the input, the user should also specify the MCQ threshold value in degrees and select the mode (sequence-independent or sequence-dependent). At the output, the algorithm provides the longest continuous segment (its location within both structures), its length and actual MCQ value. If more than one solution exists, all of them are shown to the user.

LCS-TA applies divide and conquer approach (Fig. 1) to find the optimum solution, i.e., the longest continuous segment in the model whose MCQ-based similarity to the target fragment is below the specified MCQ threshold. The computation proceeds as follows. First, the algorithm computes MCQ between entire structures. If its value does not exceed the threshold, the whole model structure is returned as the optimum solution. Otherwise, the size of the current search window is determined according to the D&C procedure described in the previous sections. Next, a set of candidate segments is constructed based on the model structure: the search window moves along the model from its 5′ to 3′-end, and all window-highlighted fragments are put into the candidate set. Thus, the current candidate set contains all segments with length equal to the current window size. After that, for every segment from the candidate set the algorithm checks if it is a feasible solution. This part of the algorithm differs between the modes. In the sequence-independent mode, the check is done by positioning the candidate segment stepwise along the target structure, i.e., the candidate segment moves along the target structure every single residue. In the sequence-dependent mode, the candidate segment is compared to the corresponding fragment of the target structure. Two sets of torsion angles, one describing the candidate and the other describing the target segment, are computed. Based on that, the MCQ value between the positioned segments is determined. If the MCQ is below the user-defined threshold, the candidate segment is a feasible solution. If the feasible solution exists in the candidate set, the algorithm tries to find the longer segment (window size is enlarged for the next iteration). Otherwise, shorter segments are considered (window size is reduced for the next iteration). The procedure iterates until the stopping condition is satisfied.

Below, we show the pseudocode of LCS-TA focusing on the general steps of the algorithm running in the sequence-independent mode. In the sequence-dependent mode, the comparison of corresponding segments is done within one *FOR EACH* loop, instead of two nested loops.
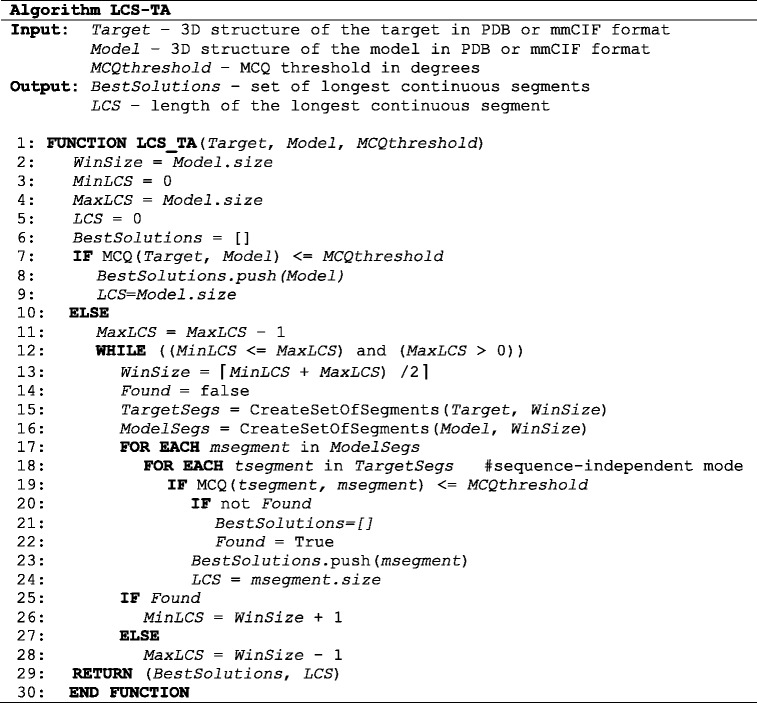


The LCS-TA algorithm in sequence-independent mode runs with the worst-case computational complexity of *O*(*n* ^*2*^log_2_ *n*). In the sequence-dependent mode the complexity is *O*(*n*log_2_ *n*), where *n* denotes the number of residues in the predicted model. This computational complexity is due to the complexity of D&C being O(log_2_ *n*), and the number of comparisons performed for every candidate segment in a single iteration.

### Accessibility and usage

LCS-TA algorithm has been implemented as a new functionality of MCQ4Structures [17], running as standalone Java Web start application. It is freely available for download at http://www.cs.put.poznan.pl/tzok/mcq/.

## Results and discussion

In this section, we present the results of LCS-TA experimental runs over selected RNA 3D structures. We analyze the algorithm’s output in the case of structure processing in sequence-independent and sequence-dependent mode, and we observe the impact of MCQ threshold value on local and global similarity assessment.

For a pair of compared RNA structures, LCA-TA algorithm provides the following output data: (i) LCS - a length of optimum solution (the longest continuous segment) measured as the number of residues in the segment, (ii) target structure coverage by the resulting segment, that is the ratio of segment to structure length (in percentages), (iii) actual MCQ value of the segment, and (iv) segment location within the structures (number of the first and last residue). If more than one optimum solution exists for two input structures, all of them are given to the user. The data are provided in plain text format and can be downloaded as CSV file.

In the first experiment, we have run LCS-TA algorithm for two RNA 3D models submitted to RNA-Puzzles challenge 18 which was compared to the target structure of exonuclease resistant RNA from Zika virus (PDB id: 5TPY) [22]. Model 1 predicted by RNAComposer [23, 24] in the server category, and model 1 submitted by Chen group [25] in the human category were selected for examination. In the paper, they are referred to as RNAComposer_1 and Chen_1, respectively. Both models were processed by LCS-TA running in two modes, sequence-independent and sequence-dependent one. In each mode, we have planned to apply the following values of MCQ threshold: 5, 10, 15, 20, 25, 30, 35 and 40 degrees. The experiment runs with MCQ threshold set to 5° returned no optimum solution for any model. On the other hand, for MCQ threshold equal to 25° the algorithm output the entire 71 nt-long structure with actual MCQ value of 23.48° in the case of RNAComposer_1, and 23.81° for Chen_1 model. This meant that MCQ of the whole model was below 25°-threshold in both cases. With 25° constituting the breakout point of the experiment no further increasing of the threshold was necessary.

**Fig. 2:**
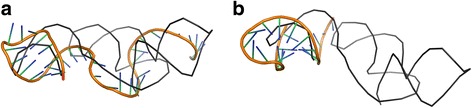
Longest segments (colored) found in sequence-independent mode, MCQ threshold = 20°, within (**a**) RNAComposer_1 and (**b**) Chen_1 models, aligned onto the target 5TPY structure (gray)

**Fig. 3:**
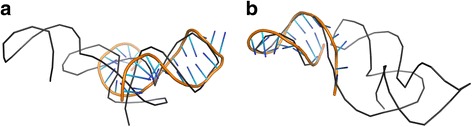
Longest segments (colored) found in sequence-dependent mode, MCQ threshold = 20°, within (**a**) RNAComposer_1 and (**b**) Chen_1 models, aligned onto the target 5TPY structure (gray)

Tables 1 and 2 present the results of RNAComposer_1 and Chen_1 models’ processing by LCS-TA with respect to the target structure in sequence-independent and sequence-dependent mode, respectively. For every MCQ threshold between 10° and 25°, we can see the position of the longest continuous segment within the model (and the target) marked with a value of 1 in the character string, segment size (LCS) and its actual MCQ value. In any case, RNAComposer_1 model dominates Chen_1, as far as LCS value is concerned. In all cases except one, the single optimum solution has been found. Only for MCQ threshold set to 10°, three segments with LCS = 9 have been identified within RNAComposer_1 model in sequence-independent mode. A closer look at the results makes us find that the most significant diversity in segment length and location within both models is observed for MCQ threshold equal to 20°. Solutions obtained for this threshold value have been visualized using PyMOL in Figs. 2 and 3. In every figure, the longest continuous segment identified in the model (colored) has been superimposed onto the target structure (grey) at the location of the corresponding target segment. As shown in the figures, different segments have been identified in the considered models.

**Table 1:**
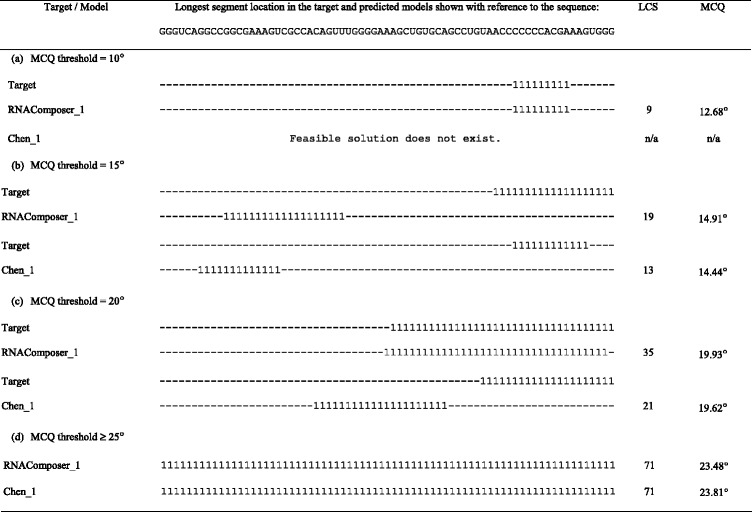
Longest segments found in the sequence-independent mode for RNAComposer_1 and Chen_1 models of 5TPY structure

**Table 2:**
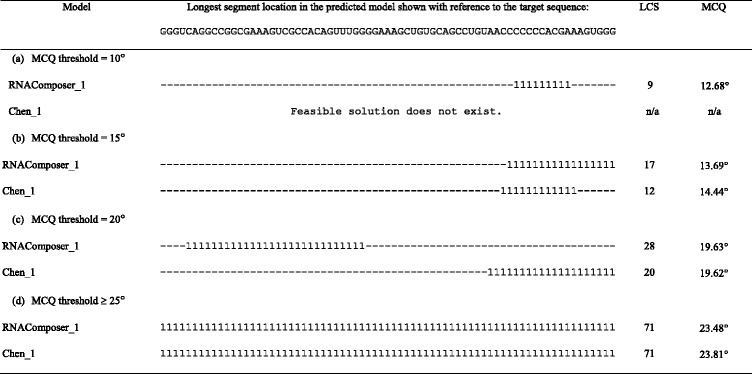
Longest segments found in the sequence-dependent mode for RNAComposer_1 and Chen_1 models of 5TPY structure

**Fig. 4:**
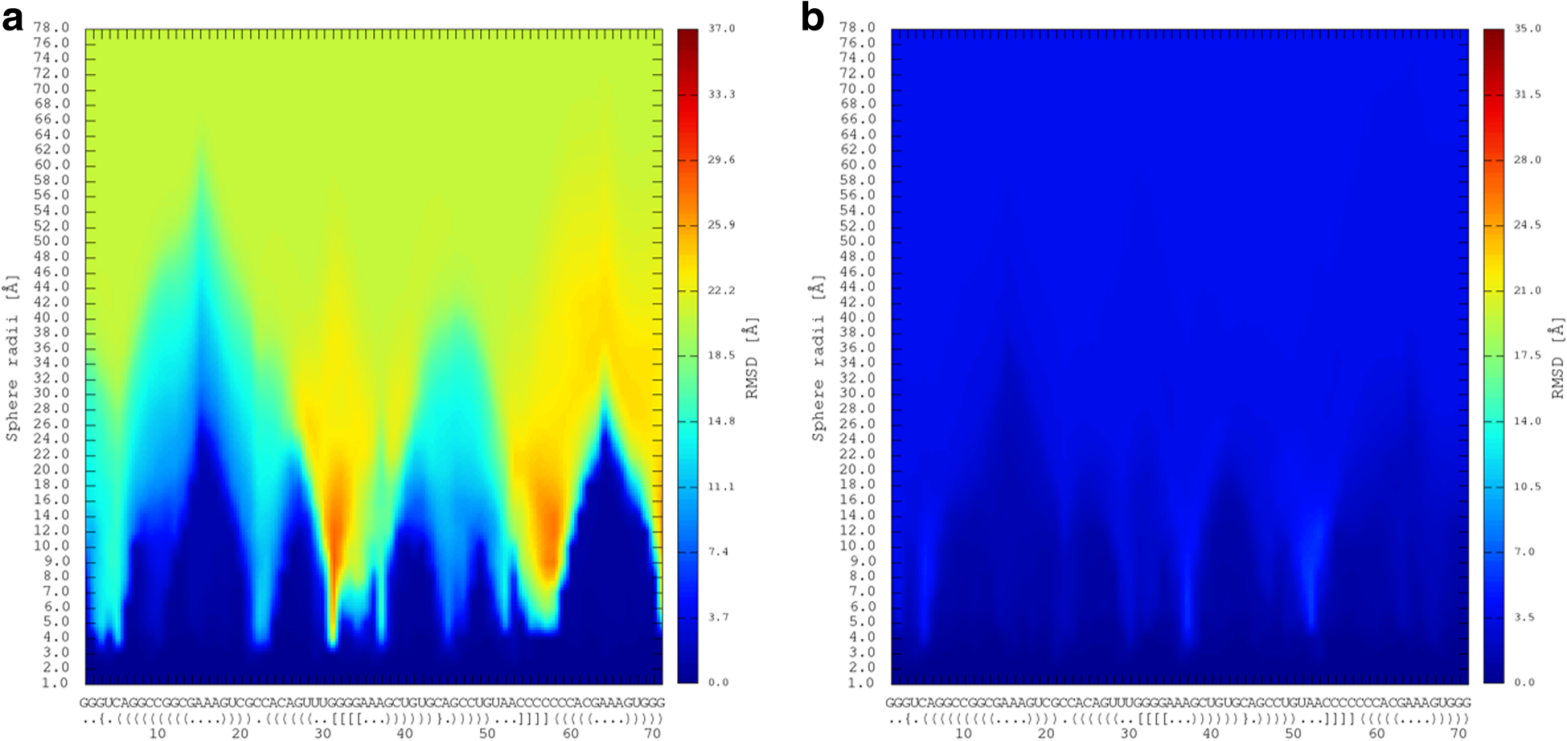
Results of (**a**) RNAComposer_1 and (**b**) Chen_1 models comparison to the target structure (5TPY) by RNAssess

To complete similarity analysis in the first experiment, we have decided to use the other similarity measure for evaluating LCS-TA results. It can be assumed that two fragments with similar torsion display the similarity also in the space of atom coordinates. Thus, to verify this assumption, we have processed RNAComposer_1 and Chen_1 models using RNAssess [19]. This tool supports the identification of local similarity between two RNA 3D structures in the sequence-dependent mode. RNAssess compares model and target structures using the idea of moving spheres and computing RMSD between RNA fragments included in the corresponding spheres (one sphere positioned in the model, the second one – in the target). The results of the comparison are provided in the graphical form (line graphs, 2D and 3D maps). To present the results of RNAComposer_1 and Chen_1 processing with reference to the target structure, we have selected 2D maps (see Fig. 4). The value of RMSD computed for sphere positioned in particular place along RNA chain is represented by colour. Dark blue areas represent fragments of high similarity. It can be observed that location of fragments identified by LCA-TA (Table 2) coincides with dark blue areas of RNAssess maps (Fig. 4). Thus, for our example structures, the similarity in torsion angle space is accompanied by the similarity in Euclidean space of atom coordinates. This is true for MCQ threshold not exceeding 20 degrees (above this threshold LCS-TA returns the whole structure as a result). Our analysis finished with computing RMSD for identified fragments of RNAComposer_1 and Chen_1 models. In the case of fragments found within RNAComposer_1 model in sequence-dependent mode, their RMSD values were equal to 0.702 Å for MCQ threshold = 10° and 0.959 Å for MCQ threshold = 15°, while the global RMSD of RNAComposer_1 equals 24.48 Å. For Chen_1 the RMSD of the LCS-TA-provided fragment was 2.011 Å for MCQ threshold = 15° (no feasible solution was found in this model for smaller threshold), while global RMSD of the model was only 3.144 Å.

**Fig. 5:**
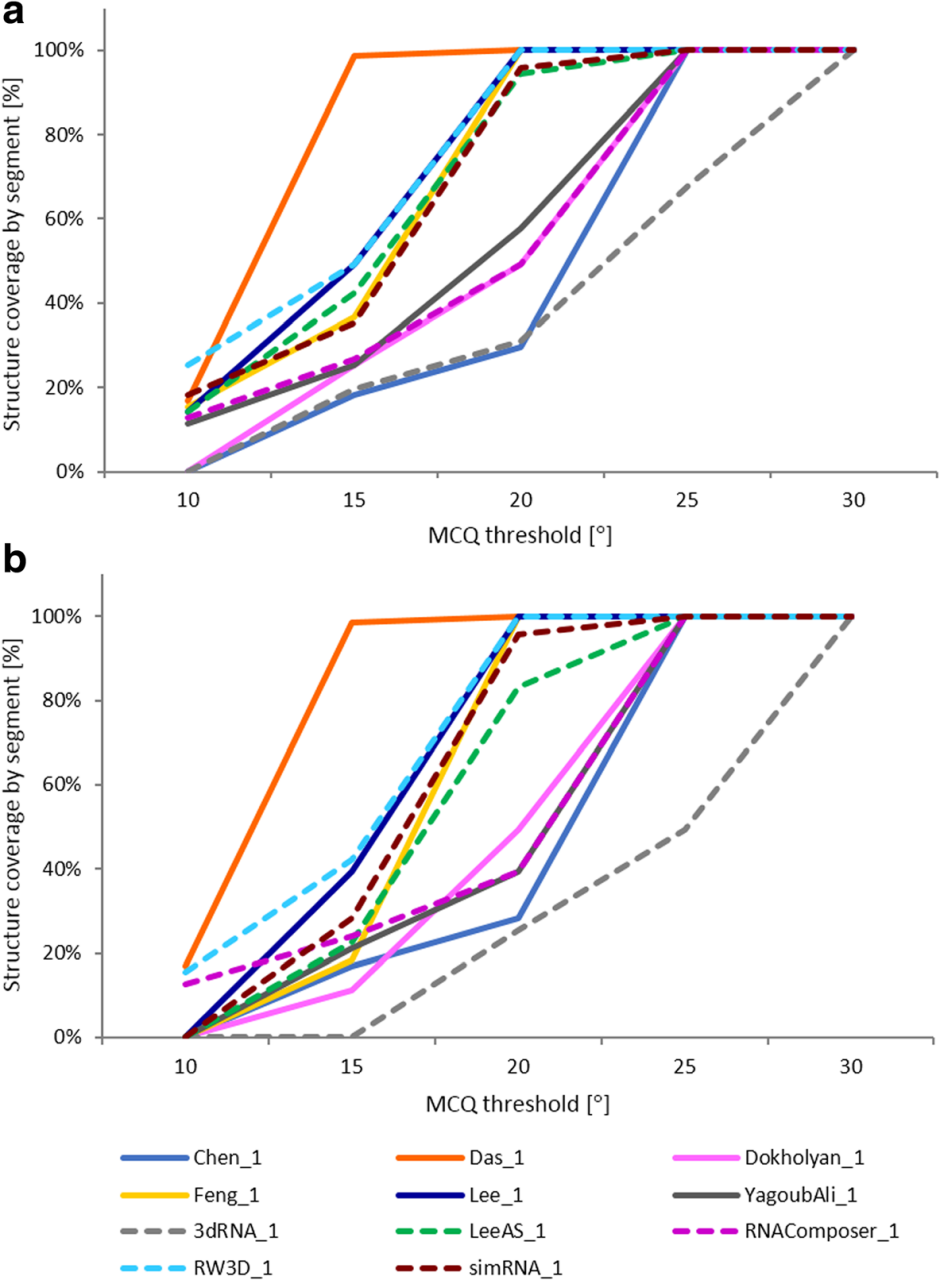
LCS-TA results for predicted models of 5TPY in (**a**) sequence-independent and (**b**) sequence-dependent mode

**Fig. 6:**
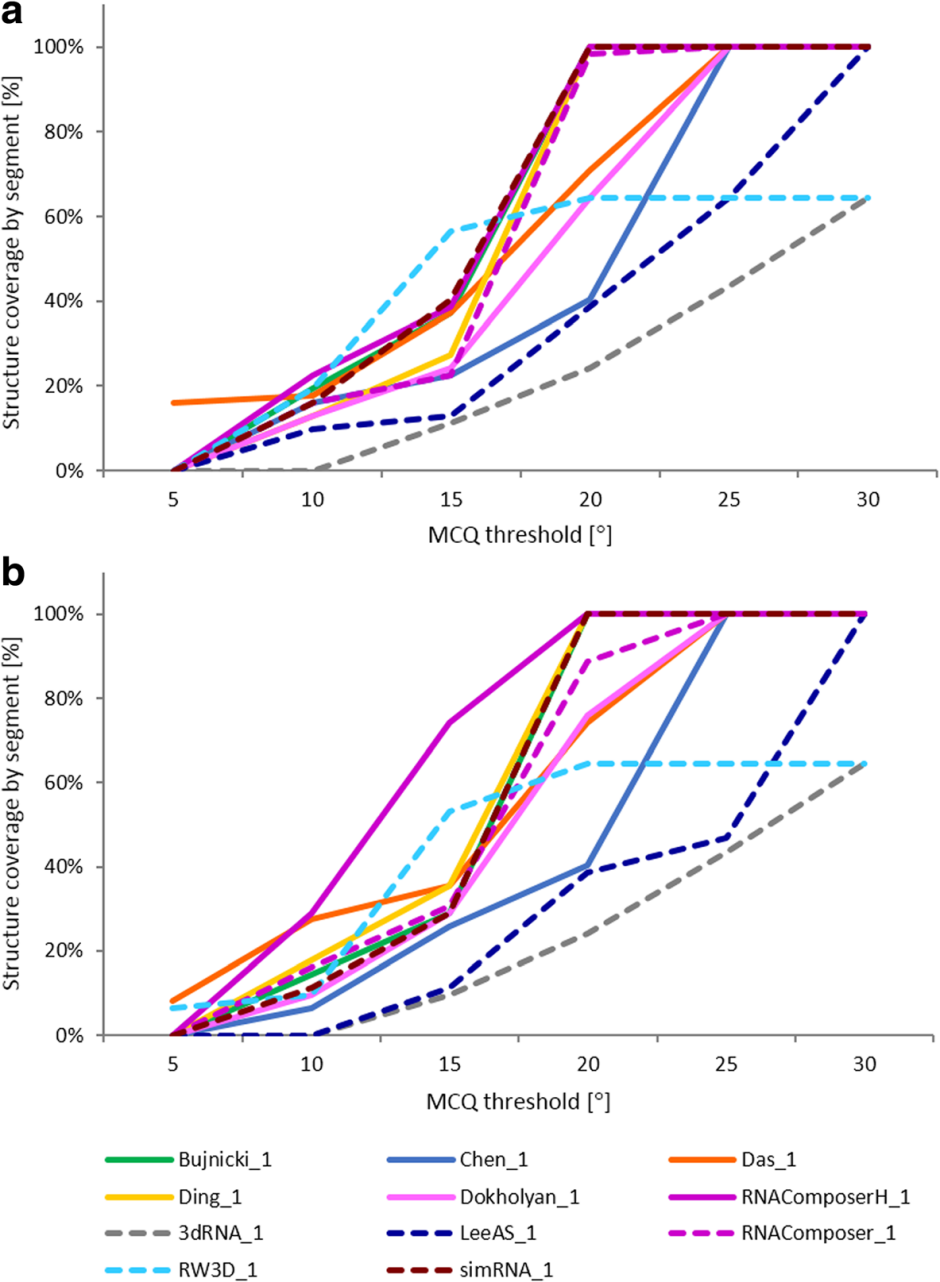
LCS-TA results for predicted models of 5T5A in (**a**) sequence-independent and (**b**) sequence-dependent mode

In the second experiment, we have investigated multiple models predicted in RNA-Puzzles challenge 18 and challenge 19. Altogether, 53 models were submitted in challenge 18, and 54 in challenge 19. From these sets, we have selected one model per each participant (namely, model 1) and we compared it to the target structure, i.e., exonuclease resistant RNA from Zika virus (PDB id: 5TPY) [22] in challenge 18, and twister sister (TS) ribozyme (PDB id: 5T5A) [26] in challenge 19. Experimental results concerning the selected models are presented in Tables 3–4 and Fig. 5 for challenge 18, and Tables 5–6 and Fig. 6 for challenge 19. In the tables, one can see LCS value, i.e., the length of the resulting segment found within each model for different MCQ thresholds, and actual MCQ of this segment. The best solution (LCS of the longest continuous segment found among all models) in human and server category is printed in bold. If more models include a segment with the biggest LCS, the one with the smallest actual MCQ is considered the winner. The figures complement tabular data by showing, for each model and MCQ threshold, the percentage of target structure covered by the optimum solution.

**Table 3:** LCS-TA results for predicted models of 5TPY structure in the sequence-independent mode

Model	MCQ threshold	10°		15°		20°		25°		≥30°	
		LCS	MCQ	LCS	MCQ	LCS	MCQ	LCS	MCQ	LCS	MCQ
(a) Human category											
Chen_1		0	n/a	13	14.80°	21	19.67°	71	23.81°	71	23.81°
Das_1		**12**	8.78°	**70**	14.98°	**71**	15.33°	**71**	15.33°	**71**	15.33°
Dokholyan_1		0	n/a	18	14.52°	35	19.40°	71	23.21°	71	23.21°
Feng_1		11	9.67°	26	14.90°	71	19.41°	71	19.41°	71	19.41°
Lee_1		10	9.83°	35	14.87°	71	18.57°	71	18.57°	71	18.57°
YagoubAli_1		8	9.70°	18	14.66°	41	19.69°	71	23.79°	71	23.79°
(b) Server category											
3dRNA_1		0	n/a	14	14.20°	22	18.58°	48	24.98°	71	26.37°
LeeAS_1		10	9.74°	30	14.99°	67	19.77°	71	20.71°	71	20.71°
RNAComposer_1		9	9.24°	19	14.91°	35	19.93°	71	23.48°	71	23.48°
RW3D_1		**18**	9.88°	**35**	14.77°	**71**	17.20°	**71**	17.20°	**71**	17.20°
simRNA_1		13	9.78°	25	14.5°5	68	19.81°	71	20.61°	71	20.61°

**Table 4:** LCS-TA results for predicted models of 5TPY structure in the sequence-dependent mode

Model	MCQ threshold	10°		15°		20°		25°		≥30°	
		LCS	MCQ	LCS	MCQ	LCS	MCQ	LCS	MCQ	LCS	MCQ
(a) Human category											
Chen_1		0	n/a	12	14.44°	20	19.62°	71	23.81°	71	23.81°
Das_1		**12**	8.78°	**70**	14.98°	**71**	15.33°	**71**	15.33°	**71**	15.33°
Dokholyan_1		0	n/a	8	13.14°	35	19.40°	71	23.21°	71	23.21°
Feng_1		0	n/a	13	14.25°	71	19.41°	71	19.41°	71	19.41°
Lee_1		0	n/a	28	15.0°0	71	18.57°	71	18.57°	71	18.57°
YagoubAli_1		0	n/a	15	14.45°	28	19.68°	71	23.79°	71	23.79°
(b) Server category											
3dRNA_1		0	n/a	0	n/a	18	19.39°	35	23.81°	71	26.37°
LeeAS_1		0	n/a	16	14.87°	59	19.89°	71	20.71°	71	20.71°
RNAComposer_1		9	9.24°	17	13.69°	28	19.63°	71	23.48°	71	23.48°
RW3D_1		**11**	9.98°	**30**	14.56°	**71**	17.20°	**71**	17.20°	**71**	17.20°
simRNA_1		0	n/a	20	14.93°	68	19.95°	71	20.61°	71	20.61°

**Table 5:** LCS-TA results for predicted models of 5T5A structure in the sequence-independent mode

Model	MCQ threshold	5°		10°		15°		20°		25°		≥30°	
		LCS	MCQ	LCS	MCQ	LCS	MCQ	LCS	MCQ	LCS	MCQ	LCS	MCQ
(c) Human category													
Bujnicki_1		0	n/a	12	8.70°	23	14.60°	62	18.92°	62	18.92°	62	18.92°
Chen_1		0	n/a	10	9.05°	14	13.53°	25	18.63°	62	22.88°	62	22.88°
Das_1		**10**	4.61°	11	8.95°	23	13.20°	44	19.72°	62	21.41°	62	21.41°
Ding_1		0	n/a	8	9.67°	17	14.44°	62	18.10°	62	18.10°	62	18.10°
Dokholyan_1		0	n/a	8	9.67°	15	14.84°	40	19.36°	62	21.42°	62	21.42°
RNAComposerH_1		0	n/a	**14**	9.56°	**24**	14.35°	**62**	18.04°	**62**	18.04°	**62**	18.04°
(d) Server category													
3dRNA_1		0	n/a	0	n/a	7	14.71°	15	19.38°	27	24.21°	40	28.16°
Lee_1		0	n/a	6	9.41°	8	14.89°	24	19.33°	40	23.97°	62	25.30°
RNAComposer_1		0	n/a	10	6.79°	14	13.00°	61	19.70°	62	20.50°	62	20.50°
RW3D_1		0	n/a	**12**	9.00°	**35**	14.66°	40	15.64°	40	15.64°	40	15.64°
simRNA_1		0	n/a	10	9.18°	25	14.64°	**62**	19.36°	**62**	19.36°	**62**	19.36°

**Table 6:** LCS-TA results for predicted models of 5T5A structure in the sequence-dependent mode

Model	MCQ threshold	5°		10°		15°		20°		25°		≥30°	
		LCS	MCQ	LCS	MCQ	LCS	MCQ	LCS	MCQ	LCS	MCQ	LCS	MCQ
(a) Human category													
Bujnicki_1		0	n/a	9	9.94°	18	14.11°	62	18.92°	62	18.92°	62	18.92°
Chen_1		0	n/a	4	9.49°	16	14.62°	25	19.85°	62	22.88°	62	22.88°
Das_1		**5**	4.91°	17	9.26°	22	14.24°	46	19.87°	62	21.41°	62	21.41°
Ding_1		0	n/a	11	9.29°	22	13.86°	62	18.10°	62	18.10°	62	18.10°
Dokholyan_1		0	n/a	6	9.61°	18	14.65°	47	19.45°	62	21.42°	62	21.42°
RNAComposerH_1		0	n/a	**18**	9.91°	**46**	14.98°	**62**	18.04°	**62**	18.04°	**62**	18.04°
(b) Server category													
3dRNA_1		0	n/a	0	n/a	6	14.63°	15	19.38°	27	24.21°	40	28.16°
Lee_1		0	n/a	0	n/a	7	12.89°	24	19.96°	29	24.48°	62	25.30°
RNAComposer_1		0	n/a	**10**	8.84°	19	14.90°	55	19.98°	62	20.50°	62	20.50°
RW3D_1		**4**	4.08°	6	8.48°	**33**	14.94°	40	15.64°	40	15.64°	40	15.64°
simRNA_1		0	n/a	7	9.24°	18	14.95°	**62**	19.36°	**62**	19.36°	**62**	19.36°

Eleven participants submitted their predictions for challenge 18. Thus, 11 RNA 3D models were selected for the analysis with LCS-TA (Tables 3–4, Fig. 5). This number includes six human predictions (Fig. 5, solid lines) and five server-predicted ones (Fig. 5, dotted lines). In the human category, the Das_1 model has appeared to win for all MCQ thresholds. Among server predictions, RW3D_1 model, generated by Das server (unpublished), has been the best. This is true for both modes of LCS-TA. In the case of sequence-independent analysis and MCQ threshold set to 10°, RW3D_1 dominates Das_1 (Table 3). However, this relationship is not the same in the sequence-dependent mode (Table 4). A comparison of the results for Das_1 and RW3D_1 with MCQ threshold = 10° in both modes shows that there is one, accurately predicted 12 nt-long segment in Das_1 which is identified by LCS-TA in both modes. However, for RW3D_1 the longest segment below 10° threshold (with LCS = 18) corresponds very well to the other part of the target structure. This influences the overall quality of RW3D_1 prediction and makes it globally a little worse than that of Das_1. Nevertheless, the accuracy and quality of both models are very high. MCQ computed for each of these models in total, does not exceed 20 degrees. Thus, starting from threshold set to 20°, the optimum solution in both cases covers 100% of the structure (Fig. 5).

Challenge 19 has also attracted 11 participants, including six in the human category (Fig. 6, solid lines) and five in the group of servers (Fig. 6, dotted lines). Thus, 11 predicted models were processed with LCS-TA (Tables 5–6 and Fig. 6). This experiment’s results show a greater diversity in the relationship between the models than in the case of challenge 18. In the human category, the situation is similar for both LCS-TA modes. Das_1 proves the best for MCQ threshold = 5°, however, when the threshold value increases by accepting values 10, 15, 20, 25 and 30 degrees, RNAComposerH_1 dominates all other models as far as LCS and actual MCQ are concerned. In the server category, the longest segments have been found in RNAComposer_1 [23, 24], RW3D_1 and simRNA_1 [27] models, depending on the MCQ threshold and LCS-TA mode. This shows that although globally the considered models seem quite similar, the differences on a local level can be significant. Thus, local analysis of the model can indicate the direction for further development and improvement of the prediction approach. From these results, we can also see that global ranking of models based on LCS-TA value highly depends on the MCQ threshold.

Molecules selected for the above analysis are medium-size RNA structures. Their processing by both alignment-based and alignment-free algorithms is possible, although it is more time-consuming in the case of the first group of methods. The difference between computing times by both groups increases significantly with the increase in molecule size. The length of RNA chain can also influence the quality of results generated by alignment-based algorithms which provide a suboptimum solution. However, this is not the case of alignment-free approach, including LCS-TA. To show that our algorithm also works for longer RNAs, we have applied it to process RNA 3D models submitted to RNA-Puzzles challenge 7 and challenge 8. In the first case, we have chosen one model per each participant (namely, model 1) and we compared it to the target structure of Varkud satellite ribozyme (PDB id: 4R4V) [28]. Similarly, the first model submitted by each participant in challenge 8 was selected and analyzed with reference to the target structure of SAM I/IV-riboswitch (PDB id: 4 L81) [29]. Altogether, we have processed seven models from challenge 7 and 6 models from challenge 8. For all cases LCS-TA algorithm provided the results, finding similar fragments positioned along the entire structure. These experiments’ results are presented in Additional file 1.

## Conclusions

In the paper, we have addressed the problem of identifying similar fragments within RNA 3D structures and tertiary structure similarity assessment on the local level. We have introduced LCS-TA method that finds fragments displaying high similarity in torsion angle space. The method has been implemented in Java and added to MCQ4Structures standalone application, freely available at http://www.cs.put.poznan.pl/tzok/mcq/. We have shown an example application of the method in processing and analysis of RNA 3D structures predicted within RNA-Puzzles challenge 18 and 19.

Our algorithm is computationally non-demanding and user-friendly. At the input, it requires PDB or mmCIF files with RNA 3D structures and MCQ threshold value. The results are easy to compare and interpret. Thus, we hope it will be of wide interest in the RNA community.

LCS-TA has the potential to open new avenues in the RNA structural bioinformatics, particularly in the field of evaluating predicted RNA 3D models, local similarity assessment, as well as in structure motif/module identification and examination. Our future works will follow in this direction. We are going to perform large-scale tests of the method to define reliable MCQ thresholds. We plan to analyze the relationship between LCS-TA results and the secondary structure motifs of the analyzed RNA structures. This kind of analysis can indicate RNA motifs or fragments which are particularly hard (or easy) to predict. Finally, we plan to supplement the algorithm with the graphical output.

## Additional file

Additional file 1: Table S1. LCS-TA results for predicted models of 4R4V structure in the sequence-independent mode. **Table S2.** LCS-TA results for predicted models of 4R4V structure in the sequence-dependent mode. **Table S3.** LCS-TA results for predicted models of 4 L81 structure in the sequence-independent mode. **Table S4.** LCS-TA results for predicted models of 4 L81 structure in the sequence-dependent mode. **Figure S1.** LCS-TA results for predicted models of 4R4V in (a) sequence-independent and (b) sequence-dependent mode. **Figure S2.** LCS-TA results for predicted models of 4 L81 in (a) sequence-independent and (b) sequence-dependent mode. **Table S5.** Longest segments found within example models of 4 L81 structure in the sequence-dependent mode. **Figure S3.** Results of (a) Bujnicki_1, (b) Das_1, and (c) Dokholyan_1 model comparison to the target structure (4 L81) by RNAssess. (PDF 465 kb)

## Abbreviations

CASP: Critical Assessment of protein Structure Prediction
CSV: Comma-Separated Values
D&C: Divide and conquer
GDT: Global Distance Test
INF: Interaction Network Fidelity
LCS: Longest Continuous Segments
LCS-TA: Longest Continuous Segments in Torsion Angle space
LGA: Local-Global Alignment
MCQ: Mean of Circular Quantities
RMSD: Root Mean Square Deviation
