# Supplementary material for: LCS-TA to identify similar fragments in RNA 3D structures
Source: BMC Bioinformatics. 2017 Oct 23;18:456. doi: 10.1186/s12859-017-1867-6 (PMC5651598; doi:10.1186/s12859-017-1867-6)

Additional file 1 (supplementary data)

LCS-TA to identify similar fragments in RNA 3D structures

Jakub Wiedemann[1], Tomasz Zok[1,2], Maciej Milostan[1,2], Marta Szachniuk[1,3*]

[1]Institute of Computing Science & European Centre for Bioinformatics and Genomics, Poznan University of Technology, Piotrowo 2, 60-965 Poznan, Poland.
[2]Poznan Supercomputing and Networking Center, Jana Pawla II 10, 61-139 Poznan, Poland.
[3]Institute of Bioorganic Chemistry, Polish Academy of Sciences, Noskowskiego 12/14, 61-704 Poznan, Poland.

*corresponding author: marta.szachniuk@cs.put.poznan.pl

Table S1: LCS-TA results for predicted models of 4R4V structure in the sequence-independent mode.

MCQ threshold / Model	5°		10°		15		20°		25°		≥30°	
	LCS	MCQ	LCS	MCQ	LCS	MCQ	LCS	MCQ	LCS	MCQ	LCS	MCQ
Adamiak_1	4	4.61°	10	8.58°	25	14.79°	25	19.52°	138	24.89°	185	26.80°
Bujnicki_1	4	4.43°	13	9.21°	**39**	14.52°	**176**	19.96°	**185**	20.33°	**185**	20.33°
Chen_1	0	n/a	6	8.64°	14	14.59°	24	19.69°	41	24.77°	75	29.95°
Das_1	**5**	4.45°	**14**	9.79°	25	14.01°	64	19.88°	185	23.09°	185	23.09°
Ding_1	0	n/a	12	9.49°	34	14.83°	91	18.98°	185	22.00°	185	22.00°
Dokholyan_1	0	n/a	10	9.63°	26	14.90°	55	19.90°	181	24.91°	185	25.43°
Major_1	0	n/a	6	9.41°	15	14.42°	23	19.80°	42	24.98°	63	29.84°

Table S2: LCS-TA results for predicted models of 4R4V structure in the sequence-dependent mode.

MCQ threshold / Model	5°		10°		15°		20°		25°		≥30°	
	LCS	MCQ	LCS	MCQ	LCS	MCQ	LCS	MCQ	LCS	MCQ	LCS	MCQ
Adamiak_1	0	n/a	7	9.72°	13	14.38°	30	19.96°	63	24.88°	185	26.80°
Bujnicki_1	0	n/a	11	9.59°	28	14.62°	**97**	20.00°	**185**	20.33°	**185**	20.33°
Chen_1	0	n/a	5	9.88°	11	14.97°	18	19.99°	29	24.99°	62	33.33°
Das_1	0	n/a	7	9.83°	14	14.85°	33	19.96°	185	23.09°	185	23.09°
Ding_1	0	n/a	**12**	9.49°	**34**	14.83°	65	19.99°	185	22.00°	185	22.00°
Dokholyan_1	0	n/a	4	9.44°	14	14.41°	23	19.58°	118	24.96°	185	25.43°
Major_1	0	n/a	0	n/a	11	14.90°	16	18.45°	28	24.53°	63	29.89°

Table S3: LCS-TA results for predicted models of 4L81 structure in the sequence-independent mode.

MCQ threshold / Model	5°		10°		15°		20°		≥25°	
	LCS	MCQ	LCS	MCQ	LCS	MCQ	LCS	MCQ	LCS	MCQ
Adamiak_1	0	n/a	13	9.47°	35	14.88°	86	19.91°	96	20.89°
Bujnicki_1	**8**	4.97°	**22**	9.41°	43	14.16°	96	17.04°	96	17.04°
Chen_1	0	n/a	8	9.65°	21	14.82°	45	19.89°	96	23.07°
Das_1	6	4.98°	18	9.87°	**87**	14.88°	**96**	15.79°	**96**	15.79°
Ding_1	0	n/a	13	9.85°	35	14.93°	95	19.53°	96	20.87°
Dokholyan_1	0	n/a	9	9.33°	18	14.17°	59	19.94°	96	22.42°

Table S4: LCS-TA results for predicted models of 4L81 structure in the sequence-dependent mode.

MCQ threshold / Model	5°		10°		15°		20°		≥25°	
	LCS	MCQ	LCS	MCQ	LCS	MCQ	LCS	MCQ	LCS	MCQ
Adamiak_1	0	n/a	8	9.49°	27	14.84°	85	19.75°	96	20.89°
Bujnicki_1	**8**	4.97°	**22**	9.41°	43	14.16°	96	17.04°	96	17.04°
Chen_1	0	n/a	0	n/a	14	14.70°	45	19.89°	96	23.07°
Das_1	0	n/a	18	9.87°	**87**	14.88°	**96**	15.79°	**96**	15.79°
Ding_1	0	n/a	6	9.96°	23	14.76°	81	19.83°	96	20.87°
Dokholyan_1	0	n/a	5	9.32°	8	14.93°	31	19.73°	96	22.42°

Figure S1: LCS-TA results for predicted models of 4R4V in (a) sequence-independent and (b) sequence-dependent mode.

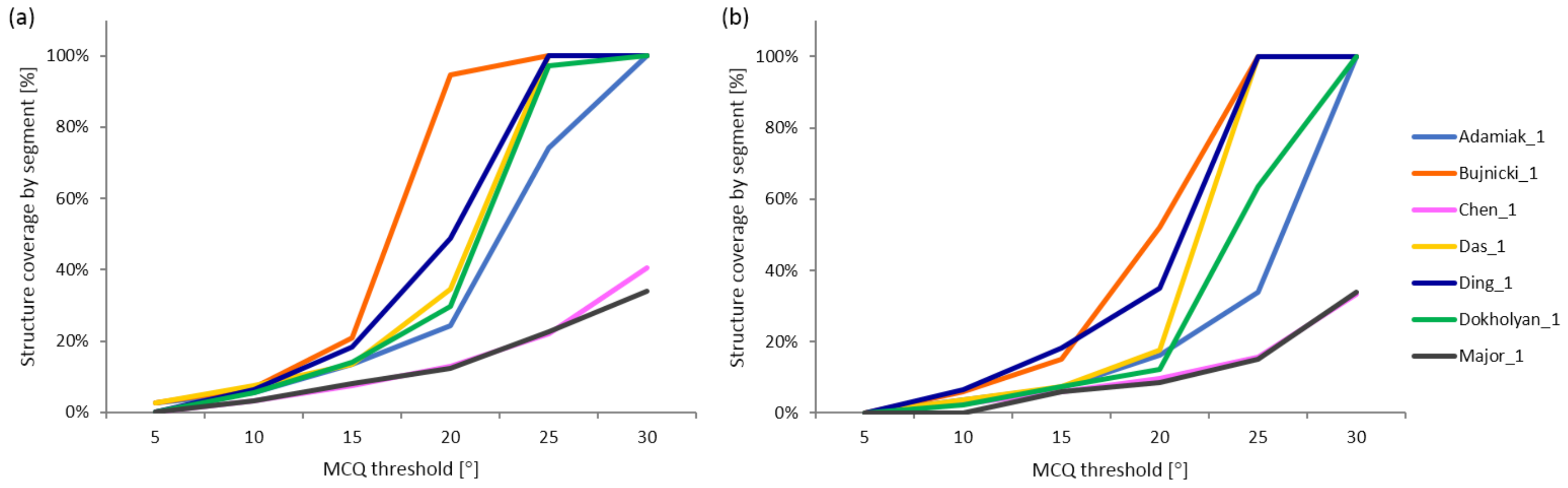

Figure S2: LCS-TA results for predicted models of 4L81 in (a) sequence-independent and (b) sequence-dependent mode.

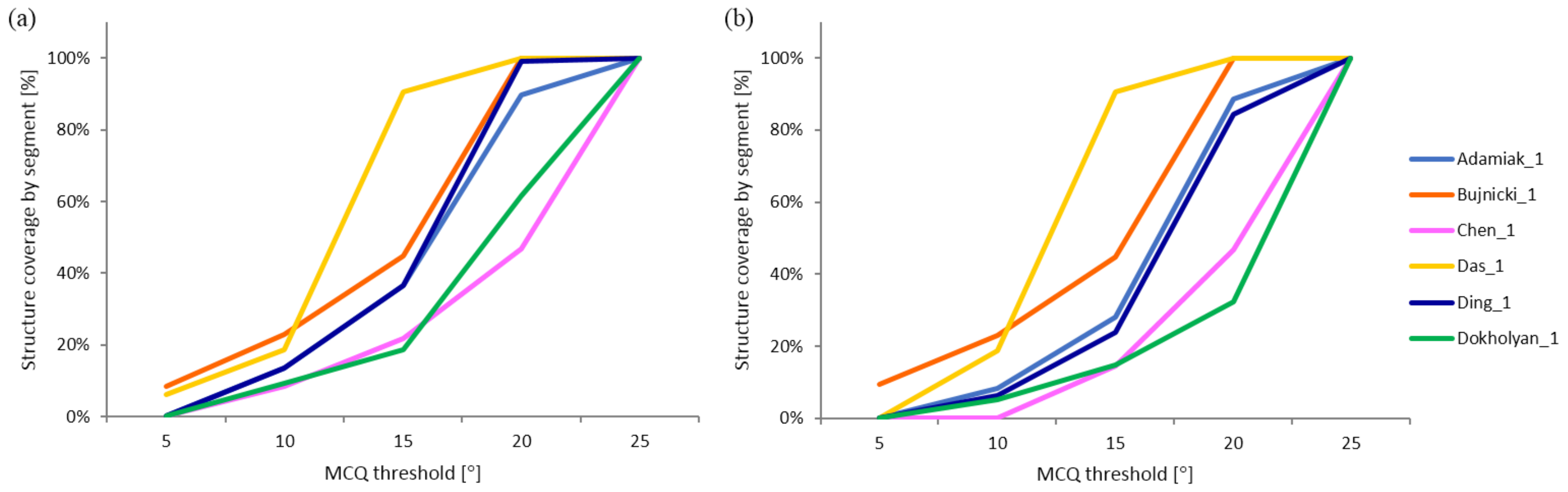

Table S5: Longest segments found within example models of 4L81 structure in the sequence-dependent mode.

Model	Longest segment location in the target and predicted models shown with reference to the sequence: GGAUCACGAGGGGGAGACCCCGGCAACCUGGGACGGACACCCAAGGUGCUCACACCGGAGACGGUGGAUCCGGCCCGAGAGGGCAACGAAGUCCGU	LCS	MCQ
(a) MCQ threshold = 5°			
Bujnicki_1	--11111111--------------------------	8	4.97°
Das_1	Feasible solution does not exist.	0	n/a
Dokholyan_1	Feasible solution does not exist.	0	n/a
(b) MCQ threshold = 10°			
Bujnicki_1	--1111111111111111111111--------------------	22	9.41°
Das_1	--111111111111111111--------------------	18	9.87°
Dokholyan_1	---11111----------	5	9.32°
(c) MCQ threshold = 15°			
Bujnicki_1	---1111111111111111111111111111111111111111111	43	14.16°
Das_1	111111111111111111111111111111111111111111111111111111111111111111111111111111111111111---------	87	14.88°
Dokholyan_1	---11111111-------	8	14.93°
(d) MCQ threshold = 20°			
Bujnicki_1	111111111111111111111111111111111111111111111111111111111111111111111111111111111111111111111111	96	17.04°
Das_1	111111111111111111111111111111111111111111111111111111111111111111111111111111111111111111111111	96	15.79°
Dokholyan_1	-------------------------------------1111111111111111111111111111111----------------------------	31	19.73°

Figure S3: Results of (a) Bujnicki_1, (b) Das_1, and (c) Dokholyan_1 model comparison to the target structure (4L81) by RNAssess.

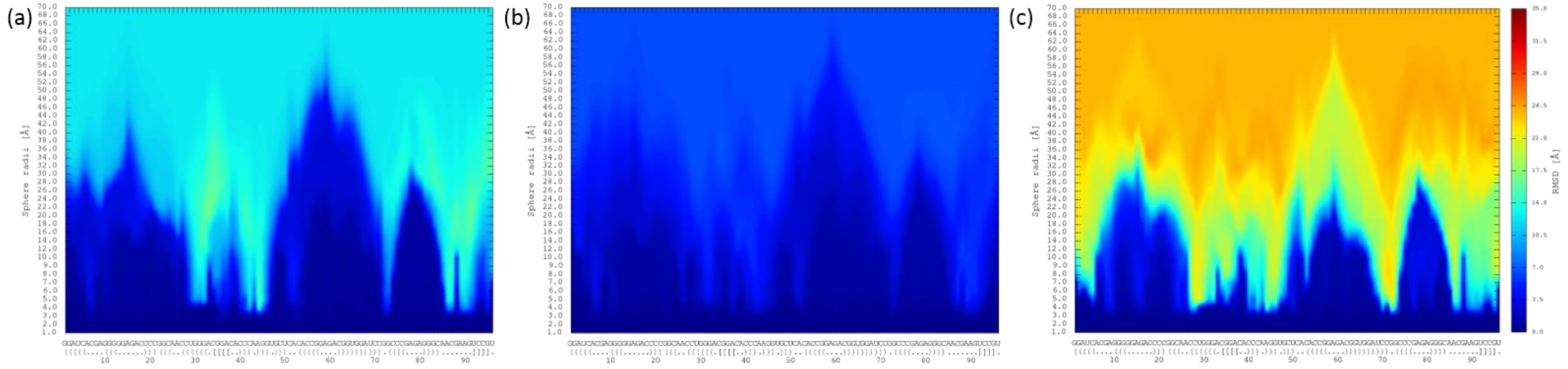

Supplement: Additional file 1: Table S1. — LCS-TA results for predicted models of 4R4V structure in the sequence-independent mode. Table S2. LCS-TA results for predicted models of 4R4V structure in the sequence-dependent mode. Table S3. LCS-TA results for predicted models of 4 L81 structure in the sequence-independent mode. Table S4. LCS-TA results for predicted models of 4 L81 structure in the sequence-dependent mode. Figure S1. LCS-TA results for predicted models of 4R4V in (a) sequence-independent and (b) sequence-dependent mode. Figure S2. LCS-TA results for predicted models of 4 L81 in (a) sequence-independent and (b) sequence-dependent mode. Table S5. Longest segments found within example models of 4 L81 structure in the sequence-dependent mode. Figure S3. Results of (a) Bujnicki_1, (b) Das_1, and (c) Dokholyan_1 model comparison to the target structure (4 L81) by RNAssess. (PDF 465 kb) [file 12859_2017_1867_MOESM1_ESM.pdf]
